# Impact of Stress on the Oral Cavity: A Cross-Sectional Study Among Dental Students

**DOI:** 10.7759/cureus.110045

**Published:** 2026-06-01

**Authors:** Meryem Rahmaoui, Fatima Ezzahra Elgasmi, Falah Nouara, Bouchra Badre

**Affiliations:** 1 Pediatric Dentistry, Mohammed VI National Center for the Disabled in Casablanca, Casablanca, MAR; 2 Odontological Sciences, Sidi Mohamed Ben Abdellah University, Fez, MAR; 3 Pediatric Dentistry, Faculty of Dental Medicine, Hassan II University of Casablanca, Ibn Rochd Hospital, Casablanca, MAR; 4 Laboratory of Community Health, Epidemiology and Biostatistics, Hassan II University, Casablanca, MAR

**Keywords:** dental education, dental students, oral health symptoms, psychological stress, stress

## Abstract

Background

Psychological stress is an important concern in dental education, as it may negatively affect students’ well-being, academic performance, and health-related behaviors. Beyond its general impact, stress may also be associated with oral health through behavioral and physiological pathways.

Methodology

A descriptive cross-sectional study was conducted among fourth- and fifth-year dental students at the Faculty of Dental Medicine of Casablanca. Data were collected using an anonymous self-administered questionnaire distributed during clinical rotations. The questionnaire explored sociodemographic characteristics, sources of academic stress related to dental training, self-perceived associations between stress and oral health, coping strategies adopted by students, and proposed measures to reduce stress within the academic environment.

Results

A total of 323 students participated in the survey, corresponding to a response rate of 94.16%. The findings indicate that a substantial proportion of students report experiencing considerable academic stress, particularly during examination periods and within demanding clinical departments. Participants self-reported several oral health complaints, including gingival bleeding, aphthous ulcers, tooth pain, and bruxism, which they perceived as being exacerbated during periods of increased stress. Students also described a variety of coping strategies, ranging from adaptive approaches, such as social support, physical activity, planning, and spiritual practices, to less adaptive behaviors, including procrastination and avoidance.

Conclusions

This study aimed to identify the main sources of academic stress among dental students, explore their perceived associations with oral health, and describe coping strategies to provide recommendations for improving student well-being and the educational environment.

## Introduction

The transition to higher education represents a critical period for young adults, marked by increased responsibilities, greater autonomy, and heightened academic pressures, all of which can generate significant stress. Stress among students is multifactorial, influenced by workload, social environment, and individual personality traits [1]. While moderate stress may enhance motivation and performance, excessive stress is associated with sleep disturbances, anxiety, and an elevated risk of burnout [1].

Dental education is particularly demanding, combining intensive theoretical instruction with early clinical exposure and increasing responsibilities toward patients. These academic constraints, compounded by personal factors, may affect not only students’ academic performance but also their mental and physical health, including oral health. International studies indicate that dental students are among the most susceptible to stress compared with peers in other academic fields, often manifesting as anxiety, fatigue, emotional exhaustion, or neglect of self-care behaviors [2-5].

In Morocco, few studies have systematically explored the sources and consequences of stress among dental students. Research at the Faculty of Dental Medicine of Monastir reported that 75% of students exhibited high psychological stress levels [6].

Although the causes of academic stress have been widely examined, its manifestations in the oral cavity remain underexplored. Recent evidence links psychological stress to oral disorders such as bruxism, gingivitis, recurrent aphthous ulcers, xerostomia, and orofacial pain. Stress may also negatively affect oral hygiene behaviors and encourage harmful habits, including increased sugar intake or smoking [7-9].

In this context, the present study aimed to identify the main stressors among fourth- and fifth-year dental students at the Faculty of Dental Medicine of Casablanca, assess their impact on oral health, and document students’ coping strategies. The study also seeks to propose recommendations to enhance student well-being within the academic environment.

## Materials and methods

This descriptive cross-sectional study aimed to assess perceived stress and associated factors among dental students at a specific point in time. The survey was conducted over a two-week period, from January 22 to February 5, 2024, and included fourth- and fifth-year students enrolled at the Faculty of Dental Medicine of Casablanca. An exhaustive sampling method was adopted, including all students registered in the two targeted academic years. Students from other academic years were excluded from the study.

Data were collected using an anonymous self-administered questionnaire adapted from a tool previously used by the Medical Student’s Trade Union during its 2011 national student health survey. The questionnaire was modified to reflect the objectives and educational context of dental training at our institution. Additional items related to preclinical and clinical dental training, organizational constraints, and self-perceived oral health effects were incorporated.

The questionnaire explored several domains, including sociodemographic characteristics; general health status; living and transportation conditions; perceived sources of stress related to theoretical, preclinical, and clinical training; self-perceived oral health problems associated with stress; coping behaviors; and students’ suggestions for stress reduction. Most items consisted of closed-ended questions using categorical response formats (yes/no questions and multiple-choice responses).

Questionnaires were distributed during hospital internship sessions according to the student rotation schedule provided by the faculty administration. Completed questionnaires were collected either at the end of the session or during the subsequent rotation to maximize participation.

Data were entered into Microsoft Excel 2007 (Microsoft Corp., Redmond, WA, USA) for coding and organization and subsequently analyzed using SPSS version 10.0 (SPSS Inc., Chicago, IL, USA). Descriptive statistical analyses were performed, and results are presented as frequencies and percentages.

## Results

A total of 323 students participated in the survey, yielding a response rate of 94.16%, including 152 fourth-year students (47.1%) and 171 fifth-year students (52.9%). The sample comprised 223 (69%) females and 98 (30.4%) males, with two (0.6%) participants not specifying their sex. Most students were first-time enrollees (n = 260; 80.5%), while 57 (17.6%) were repeaters; six (1.9%) students did not report their academic status.

Regarding general health, 302 (93.5%) students reported being in good health, while 16 (5%) reported chronic conditions; five (1.5%) participants did not respond. Most students lived with their parents (54.2%), followed by those in private housing (41.2%) and university residences (4.3%). Daily transportation to the faculty varied: 35.6% walked, 29.1% used a personal car, 20.1% took the tram, and smaller proportions used bus (6.5%), taxi (4.3%), train (2.2%), or motorcycle (1.5%). Commute times were under 15 minutes for 41.8%, 15-30 minutes for 27.2%, 30-60 minutes for 22.6%, and over one hour for 7.4% of students.

Sources of stress in dental studies

Overall satisfaction with dental training was moderate. While some students reported general satisfaction, a considerable proportion expressed dissatisfaction with workload, clinical training organization, and academic pressure. Most students frequently reported feeling overwhelmed by both theoretical and practical demands, particularly during examination periods. The theoretical curriculum was described as stressful due to the high volume of material, rapid lecture pace, and abstract content, with frequent assessments and performance expectations contributing to perceived stress.

**Figure 1:**
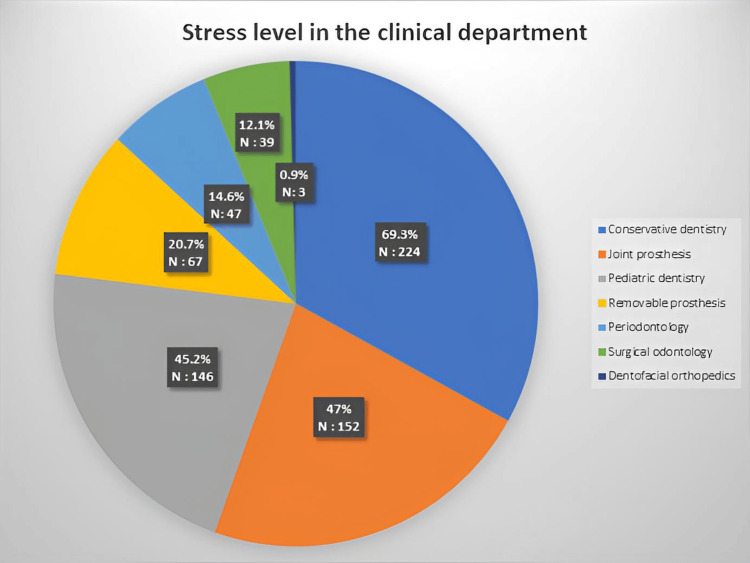
Stress level in the clinical department.

In preclinical training, conservative dentistry was identified as the most stressful department (69.3%), followed by fixed prosthodontics (47%) and pediatric dentistry (45.2%). Stress in these departments was attributed to technical complexity, continuous evaluation, and time constraints. These results are shown in Figure 1.

Stress in clinical training

Organizational factors, including delays in material availability, equipment malfunctions, and absent patients, were frequently reported as sources of stress in clinical settings. Direct patient interactions were also described as a source of pressure, with students reporting anxiety related to patient expectations, apprehension, or challenging behaviors. Fear of errors and concerns about meeting clinical requirements were also commonly reported.

Oral health problems

Many students self-reported oral health complaints such as tooth pain, gingival symptoms, and signs suggestive of bruxism, often with delayed or infrequent treatment seeking. A majority of participants perceived academic stress as being associated with oral conditions, including aphthous ulcers, gingival bleeding, and bruxism.

## Discussion

The present study confirms that dental students are particularly susceptible to academic stress, a finding consistent with previous reports in the international literature. This vulnerability arises from the cumulative demands of intensive theoretical instruction, extensive clinical training, and continuous assessments [5,9-13].

Among these, examination periods and performance evaluations were consistently identified as major stressors, highlighting the universal nature of academic pressure in dental education. Similar observations have been reported in France, Canada, and Jordan, indicating that stress associated with examinations and evaluations is a widespread challenge in health professions training [8,14-18].

Within the curriculum, clinical training was identified as the most demanding component. In our sample, conservative dentistry was perceived as the most stressful department (69.3%), followed by fixed prosthodontics (47%) and pediatric dentistry (45.2%), whereas other specialties, such as removable prosthodontics, periodontology, and oral surgery, were reported as less stressful. These findings are aligned with Pöhlmann et al., who identified endodontic care as particularly anxiety-inducing in German and Swiss dental schools [17]. The variability in stress levels across specialties suggests that both educational and cultural contexts influence students’ perceptions [19]. Importantly, stress in clinical departments was primarily attributed to organizational and material limitations, including insufficient equipment, dental chair malfunctions, and time constraints. Such conditions not only hinder clinical efficiency and autonomy but may also shift the focus from patient-centered care to performance-based validation, creating ethical and pedagogical concerns.

Time constraints and the clinical quota system were additional contributors to student stress. Nearly half of the participants reported difficulties managing their time, while 40% identified the number of required clinical procedures for validation as highly stressful. Although quotas are designed to ensure clinical competence, they may inadvertently encourage students to view patients as performance requirements rather than care recipients, potentially reducing motivation and learning satisfaction. Competency-based assessment models have been proposed as effective alternatives, allowing evaluation of clinical skills without increasing stress or compromising productivity. These findings highlight how structural and curricular factors interact with psychological stressors to impact student well-being [4,8,17,19].

Academic stress was reflected not only in psychological outcomes but also in behavioral and health-related consequences. Students reported a variety of coping strategies, ranging from adaptive approaches, such as planning, social support, physical activity, and mindfulness practices, to less effective behaviors, including procrastination, avoidance, and social withdrawal. Religious and spiritual practices were also cited as important for emotional regulation, consistent with prior studies. These findings are in line with Lazarus and Folkman’s transactional model of stress, which emphasizes the superior long-term effectiveness of problem-focused coping strategies compared with emotion-focused or avoidance-based strategies [16,20].

The impact of stress extended to students’ oral health. During periods of heightened academic pressure, participants reported xerostomia, jaw pain often associated with bruxism, recurrent aphthous ulcers, and increased gingival bleeding. Most students perceived stress as a triggering or exacerbating factor for these conditions, a perception supported by evidence linking chronic stress to elevated salivary cortisol, altered immune responses, and increased susceptibility to oral diseases. Similar associations have been reported between stress, neglect of oral hygiene, and gingival inflammation, emphasizing that academic stress can influence both psychological and physical health outcomes [2,7,10,15,21].

Student behavior in response to oral health problems also varied. Some sought prompt professional care, reflecting awareness of the importance of personal oral health in their future careers. Others delayed or neglected treatment due to time constraints, symptom minimization, or cumulative academic pressure. A minority relied on self-medication or symptomatic treatments without professional guidance, practices highlighted as potentially harmful in prior research. These findings reinforce the notion that academic stress can shape health-related behaviors and decision-making in addition to psychological functioning [13,15,22-25].

To address these challenges, students proposed multifaceted interventions targeting institutional, pedagogical, psychosocial, and individual dimensions. Recommendations included strengthening supervisory support, improving collaboration with paramedical staff, and optimizing interdepartmental coordination to facilitate clinical rotations. Psychological support measures, such as in-house counseling services staffed by trained professionals, were strongly endorsed. Extracurricular activities, including sports, cultural events, and artistic workshops, were identified as effective for promoting balance between academic and personal life, while initiatives fostering inter-cohort interactions were suggested to reduce isolation and enhance peer support. Finally, students emphasized the importance of healthy lifestyle habits, including balanced nutrition, regular physical activity, adequate sleep, and mindfulness or spiritual practices, as complementary strategies to manage stress.

Collectively, these findings underscore the multifactorial nature of academic stress in dental education, encompassing curricular demands, clinical pressures, organizational limitations, and personal coping resources. They highlight the importance of implementing integrated strategies that combine structural improvements, supportive pedagogical practices, psychological interventions, and promotion of individual coping mechanisms. Such measures may not only improve student well-being but also enhance learning outcomes and facilitate a smoother transition to professional practice [14,19,20,26,27].

This study has several strengths. First, it achieved a high participation rate, which enhances the representativeness of the findings within the targeted academic years. Second, the study adopted a multidimensional approach by exploring not only perceived academic stressors but also self-perceived oral health effects, coping behaviors, and students’ proposed stress-reduction strategies. This comprehensive perspective provides valuable insight into the complex interactions between academic demands, well-being, and health-related behaviors among dental students.

However, certain limitations should be acknowledged. The cross-sectional design only reflects perceptions at a single point in time and does not allow causal relationships to be established between stress and oral health outcomes. In addition, the study relied exclusively on self-reported data, which may be influenced by recall bias, subjective interpretation, and variability in students’ perceptions of stress and oral health conditions. The questionnaire was adapted from a previously existing survey tool rather than newly validated specifically for this population, which may affect the reliability and comparability of some findings. Furthermore, the study was conducted in a single institution, limiting the generalizability of the results to other dental schools or educational contexts. Moreover, no inferential statistical analyses were performed to quantitatively assess associations between perceived stress and oral health outcomes. Despite these limitations, the study provides important baseline data on an underexplored population and highlights key areas for future educational and psychosocial interventions.

Future research should evaluate the effectiveness of these interventions across diverse educational and cultural settings and examine their long-term impact on student performance, oral health, and psychological resilience.

## Conclusions

This study highlights academic stress as a major concern among fourth- and fifth-year dental students at the Faculty of Dental Medicine of Casablanca, with possible effects on psychological well-being, oral health, and healthcare-seeking behaviors. Stress is mainly linked to exam periods, demanding clinical rotations, especially endodontics, as well as organizational issues such as material availability, time management, and clinical requirements, and is also influenced by personal and social factors. Students use different coping strategies, and their views highlight the need for institutional actions to improve the learning environment, strengthen psychological support, and promote student well-being. However, it should be noted that these conclusions are based on a single-center, cross-sectional study using self-reported data. Therefore, the results should be interpreted with caution as exploratory and perceived associations between stress and its different aspects, without allowing causal inferences. These findings, consistent with the existing literature, support the integration of mental health considerations into dental education policies.
